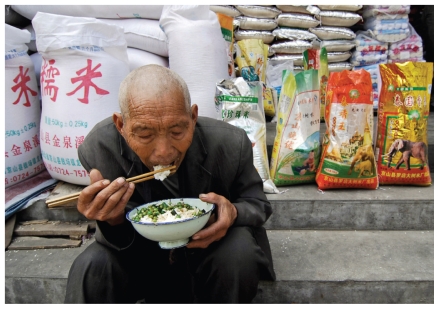# Rice Is a Significant Source of Methylmercury: Research in China Assesses Exposures

**DOI:** 10.1289/ehp.118-a398a

**Published:** 2010-09

**Authors:** Julia R. Barrett

**Affiliations:** **Julia R. Barrett**, MS, ELS, a Madison, WI–based science writer and editor, has written for *EHP* since 1996. She is a member of the National Association of Science Writers and the Board of Editors in the Life Sciences

Human activities such as mining, smelting, and coal combustion disperse mercury that can be methylated by bacteria to produce methylmercury, a potent neurotoxicant. Methylating bacteria thrive in aquatic sediments rich in organic matter, and methylmercury biomagnification eventually leads to heavy contamination of top predators, including fish consumed by humans. Although fish and seafood are the most common dietary sources of methylmercury worldwide, new research from China demonstrates that rice, a staple food for billions, can be a primary source of methylmercury in areas where there is substantial inorganic mercury pollution, with calculated exposure exceeding current tolerable daily intakes **[*EHP*** **118(9):1183–1188; Zhang et al.]**.

The research was conducted in four regions in Guizhou province, an area of inland China with rich deposits of cinnabar (a mercury ore). Mercury mining and smelting have led to heavy pollution in Wanshan, while zinc smelting and coal combustion, which also release mercury, are the main contributors in Weining and Qingzhen, respectively. The fourth region, Leigong, is a remote nature reserve selected to represent an area with no sources of direct mercury contamination.

Methylmercury and total mercury exposure through drinking water, diet, and respiration were assessed for adults in the four regions. Previous sampling provided data for air, water, fish, meat, and poultry, while agricultural products (rice, corn, and vegetables), drinking water from Wanshan and Leigong, and total gaseous mercury in Wanshan were newly evaluated in this study. These data were collectively used to calculate probable daily intakes for the general adult population.

In all regions rice, vegetables, and meat (not including poultry and fish) accounted for 89–97% of total mercury exposure, whereas rice consumption accounted for 94–96% of methylmercury exposure. Fish contributed little; most of the fish consumed here are farmed species that grow rapidly and eat a diet that precludes significant methylmercury bioaccumulation.

In Weining, Qingzhen, and Leigong, average exposures remained below provisional tolerable weekly intakes for total mercury and for methylmercury (0.57 μg/kg/d and 0.23 μg/kg/d, respectively) and a more stringent methylmercury reference dose of 0.1 μg/kg/d. However, Wanshan adults averaged 1.9 μg/kg/d for total mercury and 0.096 μg/kg/d for methylmercury. Although methylmercury represented only 5% of the total mercury exposure estimated for that area, it was enough to result in 7% of Wanshan adults exceeding the methylmercury provisional tolerable weekly intake and 34% exceeding the reference dose.

It is unknown whether methylmercury limits, which are based on fish consumption, provide adequate protection for a population with rice-based exposure because rice lacks the micronutrients found in fish that might partly offset neurotoxicity. Given that heavy inorganic mercury pollution exists in other rice-growing regions of Asia, further investigation is critical to assess exposure and correlate it with human biomonitoring (especially for pregnant women) and potential health effects.

## Figures and Tables

**Figure:**